# The developmental course of adolescent paranoia: a longitudinal analysis of the interacting role of mistrust and general psychopathology

**DOI:** 10.1007/s00787-024-02563-y

**Published:** 2024-08-29

**Authors:** Gennaro Catone, Vincenzo Paolo Senese, Antonio Pascotto, Simone Pisano, Matthew R. Broome

**Affiliations:** 1 Department of Educational, Psychological and Communication Sciences, Suor Orsola Benincasa University, Naples, Italy; 2 Department of Psychology, University of Campania “Luigi Vanvitelli”, Naples, Italy; 3 Department of Mental and Physical Health and Preventive Medicine, University of Campania “Luigi Vanvitelli”, Naples, Italy; 4 Department of Translational Medical Sciences, Federico II University, via Pansini n 5, 80120 Naples, Italy; 5 Institute for Mental Health, University of Birmingham, Birmingham, UK; 6 Birmingham Women’s and Children’s NHS Foundation Trust, Birmingham, UK

**Keywords:** Mistrust, Paranoia, Internalizing symptoms, Adolescence, Longitudinal

## Abstract

Paranoia is the erroneous idea that people are targeting you for harm, and the cognitive model suggests that symptoms increase with emotional and relational distress. A factor potentially associated with paranoia is mistrust, a milder form of suspiciousness. This study investigated the longitudinal course of non-clinical paranoia in a sample of 739 students (age range 10–12 at baseline assessment, 12–14 at second assessment) using data from the Social Mistrust Scale (SMS) and the paranoia subscale of the Specific Psychotic Experiences Questionnaire (SPEQ). Prevalence of mistrustful and high paranoia children was 14.6 and 15% respectively. Independently, baseline internalizing symptoms (b = 0.241, p < 0.001) and mistrust (b = 0.240, p < 0.001) longitudinally predict paranoia after controlling for confounders. The interaction of mistrust and internalizing symptoms at T1 increases the possibility of the onset of paranoia at T2. Therefore, the effect of mistrust on paranoia is more marked when internalizing symptoms are higher. Our results confirm the role of mistrust as a factor involved in the developmental trajectory of paranoia in adolescence, enhanced by the presence of internalizing symptoms. The implications of these results are both theoretical and clinical, as they add developmental information to the cognitive model of paranoia and suggests the assessment and clinical management of mistrust and internalizing symptoms in youth may be useful with the aim of reducing the risk of psychotic experiences.

**Supplementary Information:**

The online version contains supplementary material available at 10.1007/s00787-024-02563-y.

## Introduction

A part from belonging to the realm of schizophrenic spectrum disorders, paranoia has also been considered in the context of psychotic-like experiences (PLES), frequently found in general population and not necessarily linked to psychosis, but with a range of internalizing symptoms [1]; also, it has been recognized in the context of attenuated psychotic symptoms, observed in youth at risk for psychotic disorder (CHR-P states), as well as in other non-psychotic clinical conditions [2]. This resulted in a boost of interest about, and greater knowledge, of its risk factors and underlying cognitive and neurobiological mechanisms [3]. In the present paper, we will focus on non-clinical psychotic-like experiences, relevant to general population. In this framework, the seminal studies on the cognitive approach to paranoia represent a fundamental starting point in the attempt to grasp the phenomenology underlying the psychotic manifestations [4].

Freeman defines paranoia as the erroneous idea that people are targeting you for harm [5]. The cognitive model of paranoia implies that delusional thinking increases in the condition of interpersonal rejection sensitivity, worry and emotional distress (social vulnerability, low self-esteem) [4, 6]. Moreover, the structure of paranoia in the general population has been hypothesized as a continuous dimension consisting of four sub-classes articulated together through a process of hierarchical organization; these categories are “interpersonal sensitivity”, “mistrust”, “ideas of reference” and “ideas of persecution”. This was verified in the second British national Adult Psychiatry Morbidity Survey (APMS) sample [4] (*for additional information see the supplementary material*). Bell and O’Driscoll (2018) re-considered these findings using network analysis in the same sample finding that “worry” is the most central item of the network and that “mistrust” and “ideas of reference” are the most central sub-communities [7].

Several studies have assessed the prevalence and associated factors of paranoia in general adult populations [5, 8]. It was associated with particular aspects of life such as being single, unhappiness, poverty, poor physical health; aspects related to functioning such as lower perceived social support and cohesion, work stress and psychiatric conditions such as suicidal ideation, anxiety, worry, phobias, post-traumatic stress and insomnia with consequent increase in addictions and need for treatment [9]. Moreover, paranoia has been associated with risk of personality disorder [10]. On the other hand, studies in adolescence are scant and, in this particular maturational period, the developmental changes can lead to a greater responsiveness to the intentions of others and sensitivity to rejection [11]. In adolescence, the rate of non-clinical paranoia is high with a demonstrated association with internalizing and externalizing problems [9, 12–15] and well-being [16]. Bird and colleagues found a substantial overlap of cognitive, affective and social factors that can maintain paranoia in a 3-months longitudinal study of 33 help seeking young people aged 11–16 years [17]. They also found that paranoia is associated with negative affect, peer difficulties and several social media-related features [11]. Finally, paranoia was associated with anxiety, depression and post-traumatic symptoms and self-harm [6]. These studies constitute the milestones for the epidemiological and clinical understanding of paranoia in adolescence; however, the authors highlighted several limitations such as the presence of a small sample size and the short duration of the follow-up [17], difficulties to generalize results [11] and several sampling bias issues [6]. Especially, not all variables considered in the general cognitive model of paranoia were assessed longitudinally.

Mistrust refers to a more attenuated form of suspiciousness and it has been associated, theoretically, with vulnerability to paranoia [18]; as already noted, it is considered one of the core dimension in the hierarchical structure of paranoia [4, 7, 19]. In addition, it has been argued that, in adolescence, the presence of disproportionate mistrust can negatively shape the quality of the self-construction processes, the formation of social relationship and the success of the developmental assignments. Wong et al. firstly described mistrust in two middle childhood (8–14 years) samples, with the additional aim of testing its association with internalizing and externalizing symptoms [18]. The authors developed, validated and used the Social Mistrust Scale (SMS), a three factor (i.e., general mistrust, home mistrust, school mistrust) instrument for the developmental age. Excessive mistrust can lead to paranoid ideas and conspiracy beliefs with a scenario that can be modified over years and, therefore, it has been considered necessary to monitor the level of mistrust in the general population [20]. A step forward was represented by the findings from a study by Zhou et al., where authors used the SMS to provide data on the prevalence and heritability of social mistrust in a large population aged 8–14 years of healthy twins (n = 1512). They confirmed that mistrust is distributed along a spectrum of intensity in childhood and adolescence with mild to moderate heritability (19% total mistrust; 26% school mistrust and 40% home mistrust). Furthermore, to put mistrust into a clinical context, in a subsequent part of the paper focusing on patients with early onset schizophrenia, Zhou et al. revealed higher mistrust in patients than in controls and that the social mistrust total score correlated with positive, negative, general and total aspects of the Positive and Negative Schizophrenia (PANSS) scale [21].

From the abovementioned considerations, the aim of this study was to describe the role of mistrust and general psychopathology in the development of non-clinical paranoia. First, we verified the psychometric properties of Social Mistrust Scale and Paranoia scale in the Italian population; based on previous report [18, 22], we hypothesize, for SMS, a correlated three-factor structure comprising a general mistrust, mistrust at school and mistrust at home factors, whereas, for Paranoia scale, a mono factorial structure. Second, we described prevalence and correlates of mistrust in pre-adolescence, in terms of socio-demographics and general psychopathology. At this regard, based on Wong et al. paper, we hypothesize that mistrust, assessed at age 12–14, highly correlates to general psychopathology; we lack a specific hypothesis on gender difference, as discrepancies exist between Wong et al. and Zhou et al. papers [18, 21]. Then, having access to longitudinal data, based on the cognitive model of paranoia and previous findings [4, 11, 14], and suggestions from other authors [18, 21], we specifically hypothesized that, in the school-based cohort of early adolescents assessed in this study, both mistrust and general psychopathology predict the development of paranoid thoughts two years later in independent and interactive manner.

## Materials and methods

### Sample and procedure

This study is part of a large longitudinal, school-based project, the Bullying and Youth Mental Health Naples Study (BYMHNS), for full details, please see these references [23, 24]. Data collection was obtained throughout the administration of self-report scales to participants. In brief, twelve middle schools (with a total population of about 4445 students) joined the project, from the metropolitan and suburban area of Naples, Italy, to ensure representativeness (geographical criterion). The first wave of assessment (T1) took place in the school year 2015/2016; the second wave (T2) in the school year 2017/2018. In the first wave, we recruited a total sample of 2959 students, which represented the 66.6% of the total number of subjects that attended the schools at that period; in the second wave, we re-assessed those attending the first grade during the first wave (attending the third grade during the second wave). On a total number of 1048 subjects, 868 of them were re-assessed, that represent the 82.8% of eligible subjects. After matching T1 with T2 data and excluding some subjects with invalid or missing data, a total sample of 739 subjects will full and valid data was achieved (70.6% of eligible subjects, 371 females and 368 males, age range 10–12 at baseline assessment, 12–14 at second assessment).

To verify whether those who participated in both phases of the data collection versus those who did not (and were therefore excluded from the analyses), the two groups were compared for baseline variables. The attrition analysis comparing demographic and general psychopathology baseline variables revealed that over and above sex and age, those lost at T2 presented more externalizing symptoms at T1 (*OR* = 1.073, *p* = 0.005). No significant differences were found with regard to both internalizing symptoms (T1) or mistrust (T1).

### Measures

*Social mistrust* The Social Mistrust Scale (SMS) is a 12-item dimensional measure of childhood mistrust rated on a No (0)/Sometimes (1)/Yes (2) scale [18]. Summing all items produced a total mistrust score (out of 24); whereas summing the items on each scale three factor scores can be computed: home mistrust, school mistrust, and the general trust (reverse-scored). Examples of mistrust items include: ‘Do you feel like a target for others at home/school?’, ‘Do you think others try to harm you at home/school?’ and ‘Do you ever think that someone is following you or spying on you at home/school?’. General trust items are reverse-scored so that a higher score corresponds to higher trust: ‘Is there someone whom you can trust at home/school?’ and ‘Is there someone whom you can trust at home/school?’. In the original paper, authors reported a good alpha for total mistrust score (α = 0.70). The SMS was translated to Italian using a back translation procedure (see supplementary material). See Results section for reliability scores in our study. In this study, we assessed social mistrust at first wave of BYMHNS in 2015/16, using it as predictor of paranoia (see “Statistical analysis”). We only consider the total score of SMS in our subsequent analyses.

*Paranoia* Paranoid thoughts were assessed using the paranoia subscale of the Specific Psychotic Experiences Questionnaire (SPEQ), derived from an adaptation of the Paranoia Checklist (PC) [25], in which three items were omitted (“People communicate about me in subtle ways”, “People would harm me if given an opportunity”, “My actions and thoughts might be controlled by others”), two similar items were combined into one item (“Someone I know has bad intentions towards me” and “Someone I don’t know has bad intentions towards me” combined into one item “Someone has bad intentions towards me”) and seven items were re-worded for the specific needs of this age range. Participants responded to the 15 items on the scale on a 6-point Likert scale (0 = Not at all, 1 = Rarely, 2 = Once a month, 3 = Once a week, 4 = Several times a week, 5 = Daily). This scale was used by Ronald et al. in a general population sample of adolescents and showed very good psychometric properties (Cronbach α = 0.93, test–retest reliability across a 9-month interval *r* = 0.66, *p* < 0.001) [22]; the scale was translated to Italian and used in a clinical study of patients with anxiety disorder [13], showing good psychometric properties. See the results section for reliability scores in the present study. Here, we used paranoia scale as the outcome measure.

*General psychopathology* The Italian self-report version of the Strength and Difficulties Questionnaire (SDQ) was administered to assess general psychopathology [26]. The SDQ includes 25 items, related to five domains: (1) emotional symptoms (5 items), (2) conduct problems (5 items), (3) hyperactivity inattention problems (5 items), (4) peer problems (5 items), and (5) prosocial behavior (5 items). A score of 0, 1 or 2 is given to each item, 0 = “not true”; 1 = “somewhat true”; and 2 = “certainly true”, with a score for each subscale (range 0–10). The psychometric properties of the self-report version of the SDQ are generally good across studies [27]. For the purpose of the present study, we used an Externalizing composite score, by the sum of the Conduct Problems and Hyperactivity-Inattention Problems subscales, and an Internalizing composite score, by the sum of the Emotional Symptoms and Peer Problems subscales, both ranging from 0 to 20 [28]. Internalizing and externalizing symptoms were used as two indexes of predictors of paranoia. Cronbach alphas were 0.702 and 0.689 for the internalizing and externalizing index respectively. Internalizing and externalizing symptoms, assessed at T1, were used as predictors of paranoia.

### Data analysis

Univariate distributions of responses to each item and score were initially examined to check for missing data and normalcy [29]. In the case of missing data, the relative units were excluded since the analysis of the missing data showed percentages of 4% or less for all the items and variables. As regards the responses to the Mistrust and Paranoia scale, item statistics showed a relevant deviation from normality for almost all items (see Tables 1, 2). Therefore, the psychometric properties of both scales were investigated by means of robust statistics. Specifically, to preliminary check the dimensionality and reliability of Mistrust and Paranoia scale, confirmatory factor analyses and reliability analyses were performed. Subsequently, correlation analyses (see supplementary material) and multiple regression analyses were conducted to test hypotheses. Finally, a structural equation modeling (SEM) was tested to verify the model at the latent level (see supplementary material). All analyses were performed with R 4.2.2 software. An alpha level of 0.05 was used for all statistical tests. All reported *p-values* are two-tailed.

**Table 1:** Standardized saturations and descriptive statistics of the Social Mistrust Scale items

Item	Stem	s	k	General	Home	School
General-1	Is there someone whom you can trust at School?	2.21	3.83	0.530		
General-2	Is there someone whom you can trust at Home?	3.01	8.49	0.867		
General-3	Do people trust you with things at School?	2.09	3.38	0.562		
General-4	Do people trust you with things at Home?	2.54	5.51	0.735		
Home-1	I feel like a target for others at Home	2.67	6.53		0.695	
Home-2	Others try to harm me at Home?	2.81	7.20		0.759	
Home-3	I worry too much about others trying to get at me at Home	2.07	2.87		0.594	
Home-4	Have you ever thought that people are following you or spying on you at Home?	1.95	2.65		0.597	
School-1	I feel like a target for others at School	1.63	1.51			0.799
School-2	Others try to harm me at School?	2.43	5.04			0.815
School-3	I worry too much about others trying to get at me at School	1.30	0.32			0.745
School-4	Have you ever thought that people are following you or spying on you at School?	2.13	3.35			0.702

**Table 2:** Standardized saturations and descriptive statistics of the Specific Psychotic Experiences Questionnaire items

Item	Stem	s	k	Paranoia
Para-1	Someone has bad intentions towards me	2.32	6.60	0.787
Para-2	Bad things are being said about me behind my back	1.52	1.75	0.828
Para-3	People are being hostile towards me	2.35	5.28	0.744
Para-4	People are trying to upset me	3.01	9.80	0.779
Para-5	Someone has it in for me	1.65	2.03	0.829
Para-6	People are looking at me in an unfriendly way	2.05	3.64	0.808
Para-7	There might be negative comments being spread about me	1.52	1.87	0.821
Para-8	People might be conspiring against me	2.45	5.93	0.857
Para-9	I am under threat from others	4.67	23.56	0.839
Para-10	People are laughing at me	2.29	5.01	0.808
Para-11	People would harm me if given an opportunity	2.51	6.28	0.828
Para-12	People are deliberately trying to irritate me	2.07	3.54	0.752
Para-13	I need to be on my guard against others	2.11	3.62	0.764
Para-14	I might be being observed or followed	3.80	16.79	0.614
Para-15	I can detect coded messages about me in the press/TV/internet	3.89	16.23	0.523

*Confirmatory factor analysis* A robust confirmatory factor analysis was carried out to test the three-factor correlated structure of the 12-item version of the Mistrust scale [18] and the unidimensional structure of the 15-item Paranoia scale [22]. As fit indices, we used the maximum likelihood chi-square test (*ML*χ^2^) in combination with other statistics less affected by sample size [30]: (a) the root mean square error of approximation index (*RMSEA*); (b) the comparative fit index (CFI); and (c) the non-normed fit index (NNFI). For MLχ^2^ test values associated with *p* > 0.05 were considered well-fitting models; for the RMSEA index, values up to 0.08 or lower were considered indicating good fitting models; for the *CFI* and for the *NNFI* indices, values > 0.90 were considered as indicating adequate fit of the model to the data. Finally, the difference in MLχ^2^ statistics (MLχ^2^_diff_), and CFI (*CFI*_diff_) values were used to test the relative fit of nested models [31].

*Reliability* In order to have robust measures of the reliability of the Mistrust and Paranoia scale scales, both Cronbach’s alpha (α) and omega (ωt) for ordinal measures [32] were calculated.

*Regression analysis* To investigate the specific relationship between Mistrust and the two indices of general psychopathology, measured at T1, with Paranoia, measured at T2, and to test the interaction effect between general psychopathology and mistrust, a hierarchical multiple regression analyses was carried out. In particular, the Paranoia transformed score was regressed on sex, age, the two index of general psychopathology (i.e., internalizing and externalizing symptoms) and the mistrust transformed score. In the regression analysis, age, the two index of general psychopathology and mistrust were included as z-scores, whereas the sex was dummy coded (females = 0; males = 1). In the first step, age and sex were included; in the second step, the two index of general psychopathology were added; in the third step mistrust was added; in the fourth step the two-way interaction effects (EXT × INT, EXT × mistrust and INT × mistrust) were considered; finally, in the fifth and last step the three-way interaction effects (EXT × INT × mistrust) was considered. When the interaction effects were significant, they were studied using simple slope analysis and the Johnson and Neyman technique (JN) [33].

## Results

### Confirmatory factor analysis

*Social Mistrust Scale* In the present longitudinal dataset, results confirmed that the 3-factor correlated model had adequate fit indices, *RMSEA* = 0.035 90% CI [0.02; 0.05]; *CFI* = 0.982, *NNFI* = 0.975, MLχ^2^ (48) = 94.02, *p* < 0.001 and showed a better fit in respect to the 1-factor model, MLχ^2^_diff_ (3) = 110.50, *p* < 0.001, *CFI*_diff_ = 0.053, *RMSEA*_diff_ = 0.048. In line with the previous validation [18], results showed that all items had a saturation greater than 0.52, and that items General-1 and General-3, items Home-3 and School-3, and items Home-4 and School-4 had correlated error terms (see Table 1). Moreover, the parameter analysis showed that the three factors were highly connected (*r*s > 0.42), therefore it is conceivable to assume the existence of a higher-order dimension of mistrust and compute a total score for the scale in conjunction with sub-scale scores. This latter second-order model is an equivalent model [30] which has the same fit index as the 3-factor correlated model; for this reason, it was not tested.

*Paranoia scale* In the present longitudinal dataset, results confirmed that the 1-factor model had adequate fit indices, *RMSEA* = 0.059 90% CI [0.05; 0.07]; *CFI* = 0.981, *NNFI* = 0.977, MLχ^2^ (90) = 307.52, *p* < 0.001. In line with the previous validation [22], results showed that all items saturated on a single dimension, showing a saturation greater than 0.54 and no correlated error terms (see Table 2).

### Reliability analysis and prevalence

*Social Mistrust Scale* The scale showed a good level of internal consistency, as indicated by robust alpha and omega statistics computed either for the total score, α = 0.861 and ωt = 0.904, or for each subscale, αs > 0.743 and ωts > 0.779. In line with the previous studies [21], data confirmed that the total mistrust score was skewed, the asymmetry was 1.305 and the kurtosis was 1.182. The mean score was 2.92 (*SD* = 3.36) with a range from 0 to 16. As regards the distribution of responses, most of the children, 66.5%, had a score less than or equal to 3, and only 14.6% of the sample showed a total score greater than or equal to 7, considered a high level of suspiciousness (mistrustful children, based on Zhou et al. 2018, top 15%; 1 standard deviation above the mean).

*Paranoia scale* The total score of the scale showed a good level of internal consistency, as indicated by robust alpha and omega statistics, α = 0.954 and ωt = 0.963. Data showed that the total score was skewed, the asymmetry was 2.020 and the kurtosis was 4.684. The mean score was 9.36 (*SD* = 11.67) with a range from 0 to 72. As regards the distribution of responses, most children, 68.5%, had a score less than or equal to 9, and 15% of the sample showed a total score greater than 21.

### Regression analysis

The results of the hierarchical multiple regression (see Table 3) showed that, over and above the control variables, both the model adding the two general psychopathology dimensions (step 2), *R*^2^_diff_ = 0.082; *F*(2,760) = 32.30, *p* < 0.001, and the model adding the Mistrust measure (step 3), *R*^2^_diff_ = 0.042; *F*(1,759) = 36.83, *p* < 0.001, increased significantly the prediction of the dependent variable, paranoia. Moreover, the regression model including the two-way interaction effects (step 4), *R*^2^_diff_ = 0.011; *F*(3,756) = 3.33, *p* = 0.019, further increased the prediction of the dependent variable confirming the presence of a moderation effect between variables. The model considering the three-way interaction effect was not significant (step 5). The results obtained with the regression analysis were substantially confirmed by the SEM model which showed both the significant effect of mistrust on paranoia and the significant interaction effect between internalizing problems and mistrust on paranoia (see supplementary material).

**Table 3:** Hierarchical multiple regression analysis predicting Paranoia by sex, age, externalizing problems, internalizing problems, mistrust and their interaction (N = 765)

Predictor	*R*^*2*^_*diff*_	*B*
**Step 1**	0.011*	
Male		− 0.163*
Age		− 0.066
**Step 2**	0.082***	
Male		− 0.112
Age		− 0.053
EXT		0.073
INT		0.241***
**Step 3**	0.042***	
Male		− 0.149*
Age		− 0.035
EXT		0.021
INT		0.154***
Mistrust		0.240***
**Step 4**	0.011*	
Male		− 0.147*
Age		− 0.034
EXT		0.036
INT		0.135**
Mistrust		0.223***
EXT × INT		− 0.045
EXT × mistrust		0.043
INT × mistrust		0.084*
**Step 5**	< 0.001	
Male		− 0.147*
Age		− 0.033
EXT		0.031
INT		0.133**
Mistrust		0.217***
EXT × INT		− 0.049
EXT × mistrust		0.039
INT × mistrust		0.080*
EXT × INT × mistrust		0.013
Total *R*^*2*^	0.146***	

Paranoia: total normalized score (z-score) of the 15-item subscale of the Specific Psychotic Experiences Questionnaire; male: sex of participant dummy coding (female = 0; male = 1); age: years of participants (z-score) evaluated at T1; INT: internalizing problems (z-score) evaluated at T1 by Emotional and Peer symptoms subscales of the Strengths and Difficulties Questionnaire (SDQ); EXT: externalizing problems (z-score) evaluated at T1 by Conduct and Hyperactivity subscales of the Strengths and Difficulties Questionnaire (SDQ); Mistrust: total normalized score (z-score) of the Social Mistrust Scale evaluated at T2

***p < 0.001; **p < 0.01; *p < 0.05

Parameter analysis of the final model with main effects (step 3) revealed that, independently of the other variables in the model, the paranoia self-reported at T2 was affected in a specific way by sex, *b* = − 0.149, *p* = 0.031, self-reported internalizing problems at T1, *b* = 0.154, *p* < 0.001, and self-reported mistrust at T1, *b* = 0.240, *p* < 0.001. Over and above the other variables in the model, males reported a lower degree of paranoia, while those reporting at T1 a higher presence of internalizing problems and/or a higher degree of mistrust reported a higher degree of paranoia at T2.

**Fig. 1:**
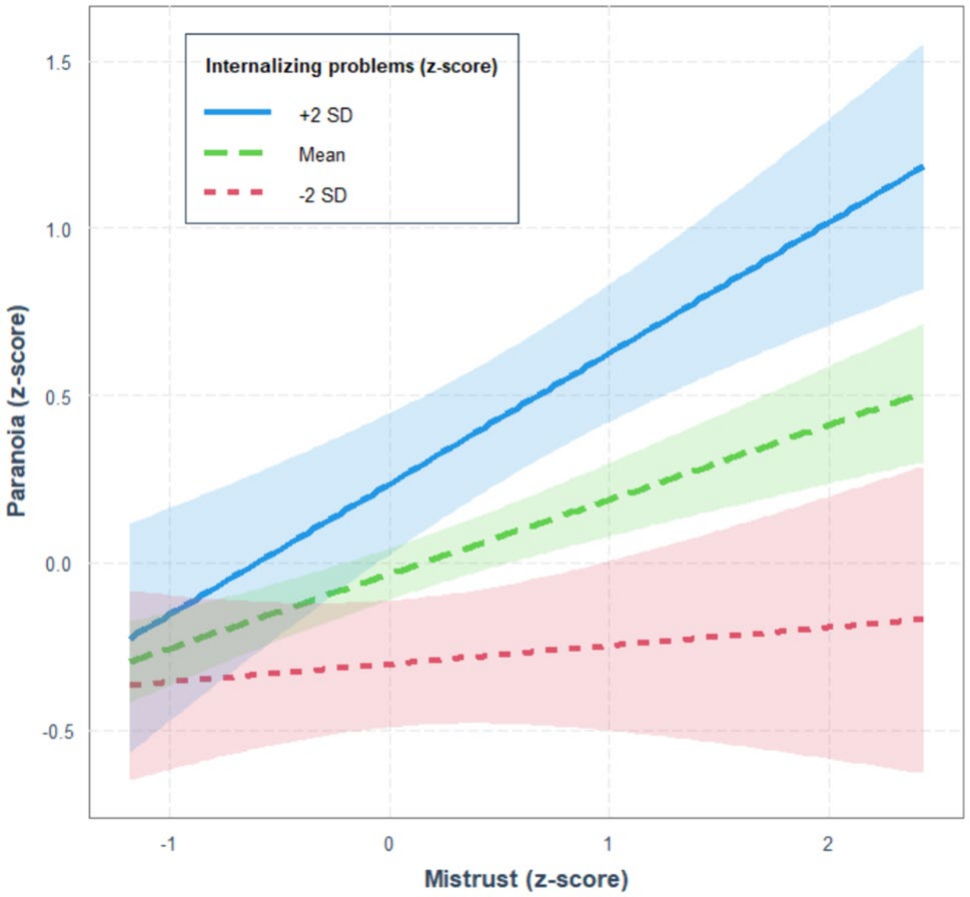
Interaction between internalizing problems, mistrust score and paranoia score

The analysis of the two-way interaction effect model (step 4) showed a significant interaction effect between internalizing problems and mistrust, indicating that the effect of each of the two variables is enhanced by the other, *b* = 0.084, *p* = 0.029. If mistrust (T1) is taken as the main variable, the moderating effect of the presence of internalizing problems (T1) is described in Fig. 1. In particular, the single slope analysis indicated that, when internalizing symptoms are low (− 2 *SD* from the mean) the effect of mistrust on paranoia is not significant, *b* = 0.054, *p* = 0.539; whereas the strength of the mistrust effect increases as the presence of internalizing symptoms increases, *b* = 0.223 and *b* = 0.391 (*p*s < 0.001), for medium or high values (+ 2 *SD* from the mean) respectively. Finally, the JN analysis indicated that the effect of mistrust on paranoia is significant when the presence of internalizing problems has an observed value greater than − 1.19 *SD* from the mean.

## Discussion

In the present paper, after confirming the psychometric properties of the related measures of interest (Social Mistrust Scale and Paranoia scale) in an Italian early-adolescence population, we analyzed longitudinally the role of mistrust and general psychopathology, in an independent and interactive manner, on later development of paranoid thinking in healthy youth.

In regard of SMS, psychometric analyses confirmed, in the Italian population, a structure of strongly correlated 3-factor model [18], with the assumption of an overdetermined factor (total score) and showed that both the total scale and the three factors had an adequate internal consistency. Similarly, in regard to paranoia scale, analyses support the previous validation of Ronald and colleagues, that revealed the 1-factor structure, with a good reliability [22]. In this regard, it is important to emphasize that although the fit of the unidimensional solution is optimal, in the absence of a comparison model for CFA, it is impossible to determine whether other solutions might better fit the data.

In cross sectional analyses, mistrust and paranoia do not differ according to age and gender and, respectively, have a strong and medium association with internalizing and externalizing symptoms. Wong et al. had already demonstrated that mistrust is less in younger children, and is associated with internalizing and externalizing problems. They also found, similarly to us, a similar rate in males and females [18].

Regarding paranoia, Bird et al. found that it was associated with worry, negative core beliefs and perceived stress. Cross sectionally, we replicate findings of a close relationship of paranoia and internalizing symptoms, assessed with SDQ. Also, we find a positive correlation with externalizing symptoms as well. This is expected, based on a previous systematic review that assessed this association (although highlighting some limitations in the quality of the studies) [34].

Our findings align with previous papers [18, 21] in reporting the distribution of mistrust in the general population to be positively skewed, with most of the children reporting low level of mistrust and a significantly smaller proportion reporting higher levels. Percentages are similar to other studies: we found 66% (compared to 50% reported by Wong et al. and 65% by Zhou et al.) reporting low mistrust (< 3 items endorsed), 14.6% reporting high mistrust (> or = 7 items endorsed) (compared to 16% reported by Wong et al. in UK sample, 18% in Hong Kong sample, 15% reported by Zhou et al. though with slightly different cut offs). Similarly for paranoia, most children, 68.5%, displayed few symptoms, whereas 15% displayed a high intensity of symptoms (greater than 21); this is similar to other epidemiological data reporting on older adolescents, extending the findings to early adolescence [22].

Our design has allowed us to construct a model in which all variables could be studied in a large sample of early adolescents in two years follow-up, addressing some of the limitations noted by Bird et al. and specifically the small sample size and low follow-up duration [11, 17, 35]. Findings demonstrated that mistrust at baseline predict future paranoia, even after controlling for general psychopathology. Subsequently, the combination of mistrust and internalizing symptoms (emotional and peer problem symptoms) at T1 increases the possibility of onset of paranoid thinking at T2. Therefore, the presence and intensity of internalizing symptoms moderates and enhances the effect of mistrust on the onset of paranoid thinking. These are results with theoretical and clinical implications. From a theoretical perspective, reflecting on the structural cognitive model of paranoia, the longitudinal element could allow us to conceive in the temporal dimension (*temporalize*) what, in the Bebbington’s model, is comprehended exclusively in the spatial dimension (*spatialize*) [4]. For Bebbington et al., the continuum of the paranoia structure can be seen, at a given point in time, as a series of differences between individuals situated at different points along the curve; we here confirmed a possible movement along the spectrum (from mistrust to paranoia), depending on developmental stage. In our opinion, our results confirmed this potential dynamic path, placing mistrust, independently, and in association with internalizing problems, as a precursor element of paranoid ideation in a developmental perspective. Whether this may confer a risk for later development of persecutory delusions, it still has to be determined, being theoretically possible.

We suggest that our findings support the conclusions of a qualitative study on the growth trajectory of paranoia in 12 adolescents (age 11–17 years). Utilizing Interpretative Phenomenological Analysis (IPA), it emerged that three moments with central themes configured the *journey* of adolescent paranoia. They were: (1) *discovering threat and vulnerability* internally depicted by a gradual (or reactive to some episodes) loss of trust in peers and uncertainty about other’s intentions; (2) *the paranoia experience* characterized by an extreme difficulty in experiencing a tendency to trust in others and a greater propensity to a sense of the other as untrustworthy; (3) *adjusting to paranoia*, a phase that is characterized by the limitation of the typical activities of adolescence and a progressive disengagement from the peer group [35].

Further, our conclusions are in line with those of Bell and O’Driscoll that indicated that the mistrust and ideas of reference were prominent aspects in maintaining paranoid state [7].

These findings cannot be directly translated to clinical settings, but they may have relevance for enhancing the early identification and prevention of psychosis and other mental disorders [31]. It is widely recognized that the study of psychotic traits and psychotic like experiences in the general population represents a valid and useful method of investigation for research questions relating to the etiology of clinical psychosis [3]. Finally, addressing mistrustful thinking, especially if associated to internalizing symptoms, should be considered central in psychotherapy for prevention of paranoia and maybe helpful to identify youth in need of more close monitoring and more tailored treatment strategy.

In addition to its merits, it is important to point out that this study has also some limitations. First of all, we did not assess paranoia at baseline. When we gathered the first wave of data the Bird et al. study [11] was not available and thus the construct of paranoia (and contextually a well validated measure of it) in pre-adolescence were still not completely elucidated. Even now, the study by Bird et al. [11] described youth aged 11 to 14 years, whereas our baseline measures were collected in youth aged 10–12 years. Hence, it could be the case that some subjects at baseline already displayed paranoid thinking (and this correlate with mistrust). Our goal, however, was to unravel a developmental precursor of paranoid thinking rather than a risk factor of a specific disease and, in this perspective, not controlling for baseline paranoia do not invalidate our results. It is worth reminding that in Italy severe mental illnesses such as schizophrenia or psychotic bipolar disorder require a special education service, that are excluded in our cohort; thus, it is highly unlikely that subjects with “clinical” delusions were included in both waves, reducing the risk of bias. It is, however, important to note that there may be an overlap with attenuated psychotic symptoms at baseline, even if the onset of clinical conditions defined in the schizophrenia spectrum is typically shifted to late adolescence or early adulthood. In this cohort, we lack some socio-environmental information (i.e. economic status, academic performance) which have been shown to be associated with paranoia and information about ethnic or sexual gender minority discrimination which represent a critical element of measuring paranoia, as they can help to disentangle the symptom from a real perceived threat [36]. Future studies should include such kind of data. Also, attrition analysis revealed that those lost to follow up displayed more externalizing symptoms than those retained, although the difference is marginal. Finally, since all variables in this study are derived from self-report measures, there is a risk of social desirability bias in the data. This means that participants may have responded in socially acceptable ways rather than truthfully, potentially compromising data validity and obscuring true variable relationships. Thus, findings should be interpreted cautiously, considering this limitation Finally, as all the variables derived from self-report measures, and so a social desirability bias cannot be excluded.

Notwithstanding these limitations, our findings put the basis for future study on the role of mistrust and internalizing symptoms in the onset of non-clinical paranoia.

## Supplementary Information

Below is the link to the electronic supplementary material.

Supplementary file1 (DOCX 243 KB)

## Data Availability

The datasets used and/or analysed during the current study are available from the corresponding author on reasonable request.
