# Supplementary material for: The developmental course of adolescent paranoia: a longitudinal analysis of the interacting role of mistrust and general psychopathology
Source: Eur Child Adolesc Psychiatry. 2024 Aug 29;34(4):1415–24. doi: 10.1007/s00787-024-02563-y (PMC12000268; doi:10.1007/s00787-024-02563-y)
Supplement: Supplementary file 1 — Supplementary file1 (DOCX 243 KB) [file 787_2024_2563_MOESM1_ESM.docx]

**SUPPLEMENTARY MATERIAL**

1 SUPPLEMENTARY INTRODUCTION

The Adult Psychiatric Morbidity Surveys (APMS) investigated data on the prevalence of psychiatric disorders in the adult population (aged 16 +) and change in these data over time. There are four subsequent surveys in 1993, 2000, 2007 and 2014. The second APMS (2007) was conducted by the National Centre for Social Research (NatCen) with the University of Leicester, upon request of The NHS Information Centre for health and social care. The survey included two-phases. The first phase contained systematized diagnostic evaluations and screening activities for a variety of mental disorders. The second phase included interviews from trained clinical researchers. The sampling was obtained using the Small Area Postcode Address File with socio-economic stratification. The total sample of individuals who completed phase 1 consisted of 7403, 849 adults were designated for the phase two of the survey. Phase two interviews were conducted with 630 of these (74%).

2 SUPPLEMENTARY DATA ANALYSIS

*Correlation analysis.* To analyse the degree of association between the variables considered, a correlation analysis was conducted. Since the variables mistrust and paranoia presented problems of normality of the distribution of values (see Table 3), the association was estimated using Spearman correlation coefficient. Finally, given the large sample size, the Hommel correction to the correlation coefficient *p-values* was applied to limit the risk of type I error (Hommel, 1988).

3 SUPPLEMENTARY RESULTS

*Correlation analysis*

The table shows the main descriptive statistics of the variables considered and the results of the correlation analyses (see Table 3). Regarding the control variables (sex and age), evaluated at T1, data showed only an association between sex and internalising symptoms, confirming that males reported fewer internalising symptoms than women. By contrast, the relationship with the other variables and with paranoia (measured at T2) was not significant.

As for general psychopathology (measured at T1), the results showed a strong and positive association between the two types of symptoms (internalizing and externalizing), sharing about 27% of the variance, that both dimensions were strongly associated with the mistrust measure (measured at T1), and showed a medium association with the paranoia measure (measured at T2) –with internalizing symptoms more associated than externalizing symptoms, *z-test* of the difference = 2.02, *p* = .04. Thus, greater mistrust and paranoia are associated with higher general psychopathology. Finally, as regards the relationship between mistrust (T1) and paranoia (T2), the results showed a medium association. Thus, greater paranoia is associated with greater mistrust.

**INSERT TABLE 1 ABOUT HERE**

**Table 1**

*Descriptive statistics and Spearman correlations coefficient between considered variables*

| Variables | 1 | 2 | 3 | 4 | 5 | 6 | 7 | 8 | 9 | *M* | *SD* | *s* | *k* |
| --- | --- | --- | --- | --- | --- | --- | --- | --- | --- | --- | --- | --- | --- |
| 1. Male |  |  |  |  |  |  |  |  |  | − | − | − | − |
| 2. Age | -.019 |  |  |  |  |  |  |  |  | 10.86 | 0.49 | 0.09 | 3.07 |
| 3. EXT | .039 | .017 |  |  |  |  |  |  |  | 5.21 | 3.30 | 0.69 | 0.37 |
| 4. INT | -.110^*^ | -.032 | .522^***^ |  |  |  |  |  |  | 4.72 | 3.54 | 0.97 | 0.57 |
| 5. SDQ | -.039 | -.006 | .870^***^ | .862^***^ |  |  |  |  |  | 9.92 | 5.99 | 0.80 | 0.44 |
| 6. Mistrust | .030 | -.086 | .426^***^ | .459^***^ | .513^***^ |  |  |  |  | 2.92 | 3.36 | 1.30 | 1.18 |
| 7. Mistrust General | .062 | -.072 | .268^***^ | .305^***^ | .328^***^ | .637^***^ |  |  |  | 0.83 | 1.38 | 1.93 | 3.66 |
| 8. Mistrust Home | -.042 | -.038 | .334^***^ | .326^***^ | .383^***^ | .735^***^ | .227^***^ |  |  | 0.88 | 1.37 | 1.92 | 4.08 |
| 9. Mistrust School | .006 | -.082 | .389^***^ | .432^***^ | .477^***^ | .814^***^ | .286^***^ | .539^***^ |  | 1.21 | 1.74 | 1.61 | 2.12 |
| 10. Paranoia | -.092 | -.072 | .198^***^ | .267^***^ | .270^***^ | .307^***^ | .164^***^ | .231^***^ | .324^***^ | 9.36 | 11.67 | 2.02 | 4.68 |

*Note*. Male: sex of participant dummy coding (female = 0; male = 1); Age: years of participants; EXT: Externalizing problems evaluated at T1 by Conduct and Hyperactivity subscales of the Strengths and Difficulties Questionnaire; INT: Internalizing problems evaluated at T1 by Emotional and Peer symptoms subscales of the Strengths and Difficulties Questionnaire; SDQ: psychological adjustment evaluated at T1 by Strengths and Difficulties Questionnaire total score; Mistrust: general mistrust evaluated at T1 by Social Mistrust Scale total score; Paranoia: paranoia evaluated at T2 by 15-item subscale of the Specific Psychotic Experiences Questionnaire total score; *Hommel’s corrected p-value < .05; ***Hommel’s corrected p-value < .001.

**Table 2**

*Italian translation of the Social Mistrust Scale*

| **Item** | **Stem** |
| --- | --- |
| General-1 | C'è qualcuno di cui ti puoi fidare a scuola? |
| General-2 | C'è qualcuno di cui ti puoi fidare a casa? |
| General-3 | Qualcuno si fida di te a scuola? |
| General-4 | Qualcuno si fida di te a casa? |
| Home-1 | Mi sento bersaglio di altri a casa. |
| Home-2 | Qualcuno prova a farmi del male a casa? |
| Home-3 | Mi preoccupo molto che altri tentino di vendicarsi a casa. |
| Home-4 | Hai mai pensato che alcune persone ti stanno seguendo o spiando a casa |
| School-1 | Mi sento bersaglio di altri a scuola. |
| School-2 | Qualcuno prova a farmi del male a scuola ? |
| School-3 | Mi preoccupo molto che altri tentino di vendicarsi a scuola. |
| School-4 | Hai mai pensato che alcune persone ti stanno seguendo o spiando a scuola. |

**Figure 1**

*Measurement model of the Social Mistrust Scale*


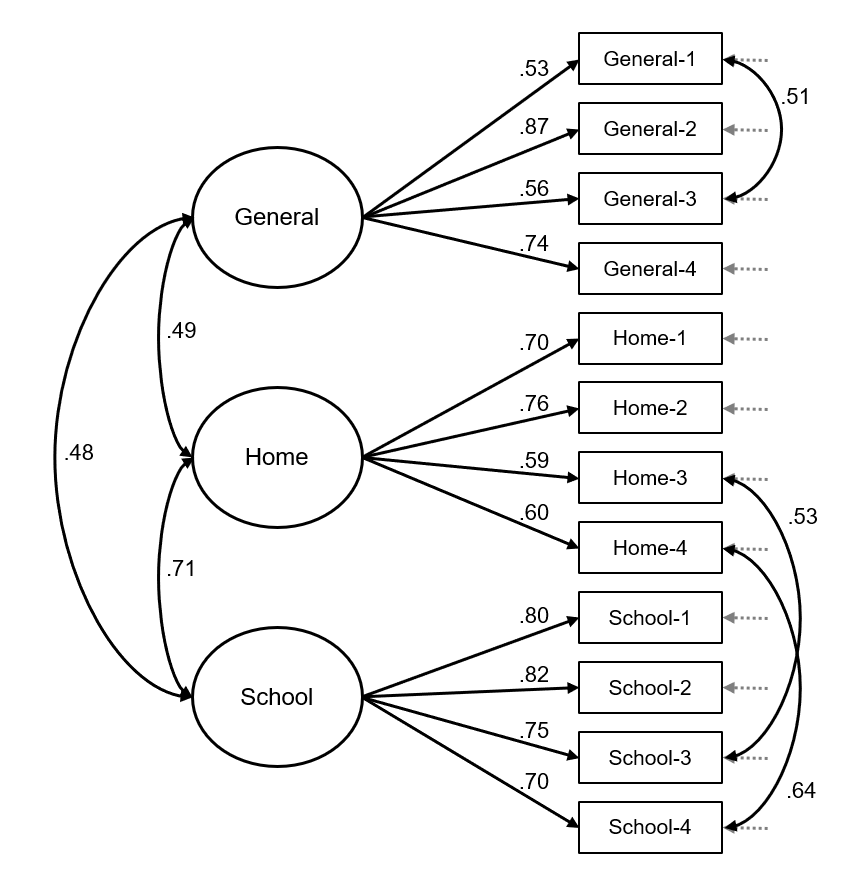


*Structural model (SEM)*

The structural model depicted in the Figure 2 and developed according to the results of the multiple regression analysis confirmed that also at the latent level, paranoia was significantly predicted by mistrust and by the interaction between mistrust and internalizing problems, *ML*χ^2^(119) = 510.03, *p* < .001, *CFI* = .921, *RMSEA* = .068, 90% *CI* = .06-.07, *SRMR* = .103. Although the interpretation does not change, it is worth noticing that, unlike the regression model, in the SEM model, the main effects of the sex and the internalizing problems were not significant.

**Figure 2**

*SEM model showing the direct effect of mistrust on paranoia and the significant interaction effect between internalizing problems and mistrust on paranoia*


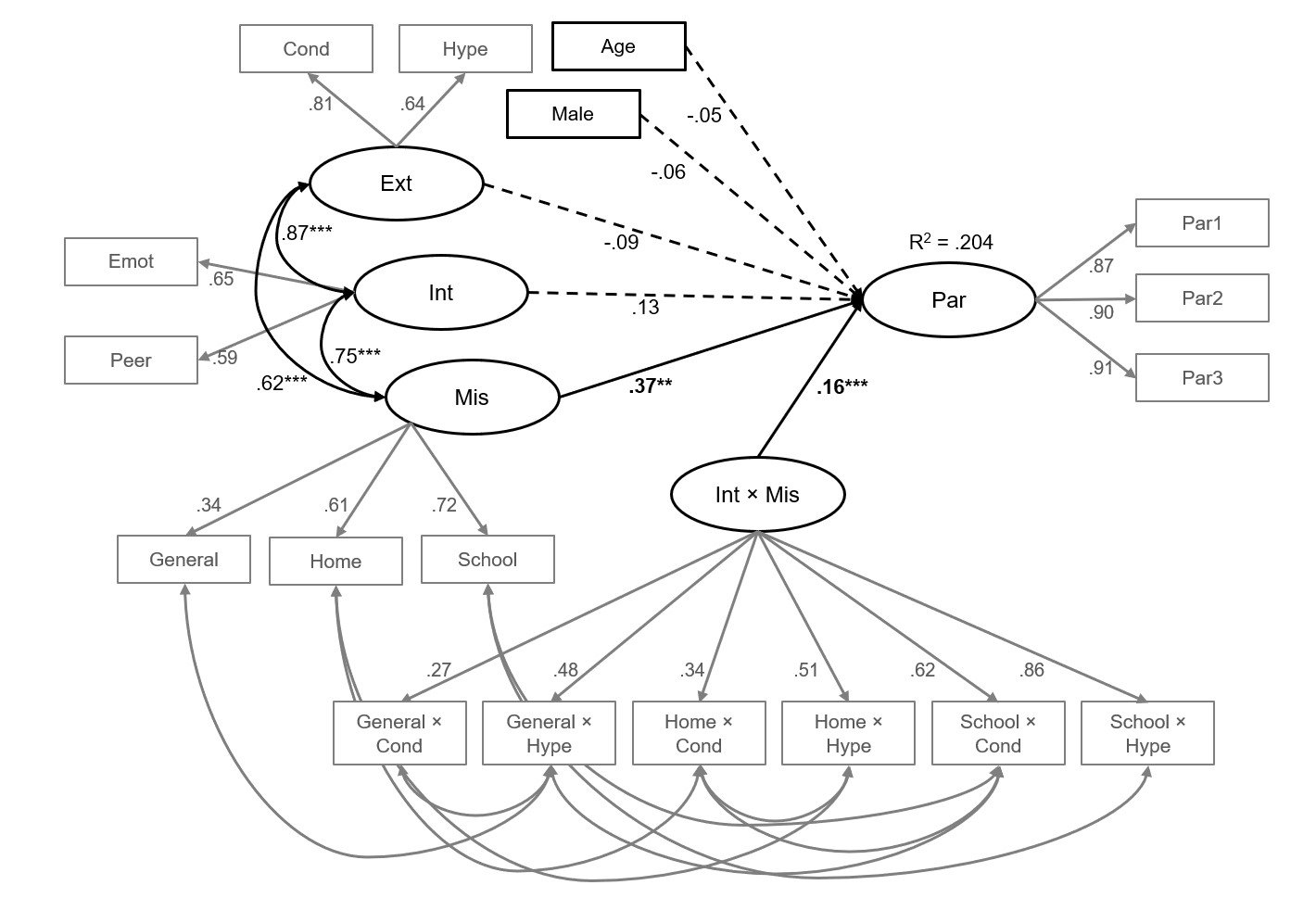


*Note*. Male: sex of participant dummy coding (female = 0; male = 1); Age: years of participants; Ext: latent Externalizing problems evaluated at T1 by Conduct (Cond) and Hyperactivity (Hype) subscales of the Strengths and Difficulties Questionnaire; Int: latent Internalizing problems evaluated at T1 by Emotional (Emot) and Peer symptoms (Peer) subscales of the Strengths and Difficulties Questionnaire; Mis: latent mistrust evaluated at T1 by General, Home and School subscales of the Social Mistrust Scale; Int × Mis: latent interaction effect between internalizing problems and mistrust on paranoia evaluated at T1 by double-mean-centred interaction terms; Para: latent paranoia evaluated at T2 by considering three parcels (Para1, Para2 and Para3) from the 15-item subscale of the Specific Psychotic Experiences Questionnaire; **p* < .05; ***p* < .01; ****p* < .001.

Additional reference list

Hommel, G. (1988). A stagewise rejective multiple test procedure based on a modified Bonferroni test. *Biometrika*, *75*(2), 383-386.
